# Editorial: Sepsis in Neonates and Children

**DOI:** 10.3389/fped.2020.621663

**Published:** 2020-11-27

**Authors:** Eric Giannoni, Luregn J. Schlapbach

**Affiliations:** ^1^Department Mother-Woman-Child, Clinic of Neonatology, Lausanne University Hospital and University of Lausanne, Lausanne, Switzerland; ^2^Child Health Research Centre, The University of Queensland, Brisbane, QLD, Australia; ^3^Paediatric Intensive Care Unit, Queensland Children's Hospital, Brisbane, QLD, Australia; ^4^Pediatric and Neonatal Intensive Care Unit, Children‘s Research Center, University Children’s Hospital Zurich and University of Zurich, Zurich, Switzerland

**Keywords:** neonate, child, immune dysregulation, sepsis, septic shock, mortality, shock

## Introduction

Sepsis, defined as life-threatening organ dysfunction resulting from dysregulated host response to infection, affects over 25 million children every year, causing an estimated 3 million deaths in neonates, children and adolescents globally (1). The life-time incidence of sepsis is strongly age-dependent, with highest rates observed in preterm neonates, followed by neonates, infants, and children (2).

Specific challenges have traditionally hindered progress in the field of sepsis in children (3). Albeit epidemiology is often split into neonatal and pediatric age groups, the evidence to support the traditional 1-month-of-age cut-off stands on shaky grounds. Sepsis in very preterm neonates exposed to multiple iatrogenic risks likely represents a very distinct disease from vertically transmitted early-onset-sepsis in a term newborn, pneumococcal sepsis in a young infant, hospital-acquired sepsis in a neutropenic child, or staphylococcal toxic shock in an adolescent patient. Accurate characterization of the disease remains problematic, even if the Sepsis-3 concept of suspected or confirmed infection with organ dysfunction is in principle applicable to the pediatric and neonatal population (4). Similar to adults, there is a discrepancy between the global distribution of sepsis burden and the high income settings where the majority of sepsis research has been performed. In addition, ethical challenges relating to consent processes and enrolment of critically ill patients can represent obstacles to conduct interventional studies, and blood sampling availability for research faces particular challenges in young children. Finally, in 2020, too many children receive antibiotics, but too many still die from infection. We have to emphasize the importance of early detection and risk stratification, prompt administration of antimicrobials, rapid resuscitation, and supportive care for organ dysfunction (5).

The aim of the Frontiers in Pediatrics Research Topic “Sepsis in Neonates and Children” was to collect state-of-the art articles and reviews highlighting the challenges, obstacles, and opportunities in assessing sepsis burden, understanding sepsis, and improving sepsis outcomes in neonates and children. We hereby provide an overview of this Frontiers in Pediatrics topic which includes 10 original articles, 9 review articles, 2 systematic reviews, and 1 perspective article.

## Pathophysiology of Sepsis: New Insights and Clinical Implications

The autonomic nervous system (ANS) regulates the functions of many organ systems, responding to stressors such as infection. Badke et al. reviewed the evidence supporting the key role of the ANS response to infection, and the importance of ANS dysfunction in the pathophysiology of sepsis. The ANS is activated early on during infection by afferent fibers which sense pathogens and tissue damage. Such activation can be assessed by changes in heart rate variability which have been shown to be associated with organ dysfunction and death in adults and children. Hence, non-invasive monitoring of heart rate characteristics represents a promising early warning tool to detect sepsis, and is associated with reduced mortality in preterm newborns (6).

Sensing of pathogens and tissue damage leads to rapid alterations of innate and adaptive immune responses, complement and coagulation, vascular, neuronal, metabolic, and endocrine systems. The early peak mortality in sepsis is associated with overwhelming inflammation and organ dysfunction seen across all age groups. At the same time, sepsis-induced immune suppression, a state characterized by exhaustion of innate and adaptive immune responses has been described in adults, leading to impaired pathogen clearance, reactivation of latent viral infections, nosocomial infections and late mortality (7). A limited capacity to mount efficient immune response mediates the increased susceptibility to infection observed in newborns (8) and is likely affected by suppression by immune cells, erythroid cells, and placental mediators. Contrary to the traditional belief that the neonatal immune system is primarily characterized by anergy or low function, newborns can in fact display dysregulated immune responses associated with excessive inflammation and early death (9). Hibbert et al. reviewed the evidence suggesting that sepsis may induce immune suppression in neonates or aggravate a preexisting state of developmental immune suppression. A better understanding of the biological pheno-or endotypes of newborns and children with sepsis will open avenues for future immune modulating strategies.

Gestational age and postnatal maturation are important determinants of the developmental state of immune responses, with the evolving microbiome and interaction with host nutrition having a strong influence (10). Schüller et al. reviewed distinct phenotypes of the developing neonatal immune system, and the immunological characteristics that may be implicated with the increased susceptibility to infection observed in early-life. They review immune modulating therapies to prevent and treat neonatal sepsis and emphasize the importance of human milk to prevent neonatal and infant sepsis (11, 12). This field is rapidly evolving, as illustrated by current studies to optimize dose and test efficacy and safety of pentoxifylline in treatment of neonatal sepsis (Clinical Trials NCT04152980, ACTRN12616000405415). The findings presented should be considered as well in view of meta-analyses on enteral supplementation with probiotics to reduce rates of NEC, late-onset sepsis and all-cause mortality in very preterm newborns (13). While evidence for efficacy is strong, there are still open questions regarding the selection of probiotic strains, dose, duration of treatment, and quality control of available products (14). In this context, the study by Esaiassen et al. evaluates the influence of probiotics and antibiotics on the developing gut microbiota and its antibiotic resistome, defined as the collection of all antibiotic resistance conferring genes. In their observational study, preterm infants <28 weeks supplemented with probiotics had a higher exposure to antibiotics compared to non-supplemented preterm (28–31 weeks) and term infants. Interestingly, microbial diversity and resistomes were not different between the three groups, which may be interpreted that the probiotic strains reduce the harmful effects of antibiotics on gut microbiota composition and antibiotic resistome development.

## Early Diagnosis and Risk Stratification

In adults, Sepsis-3 differentiates sepsis from uncomplicated infection by the presence of organ dysfunction (15). The Sequential Organ Failure Assessment (SOFA) score has better prognostic accuracy in adults compared to former sepsis criteria. Age adapted pediatric (pSOFA) and neonatal (nSOFA) scores have shown promising results in pediatric intensive care units (PICUs) and neonatal intensive care units (NICUs) from high-income countries. Yet applicability to global settings remains controversial (16–18). Obonyo et al. discuss the challenges to apply sepsis criteria to children cared for outside intensive care, including emergency department (ED) and in low-and-middle-income countries. The systematic review by Liang et al. on clinical risk factors for mortality in neonates and infants hospitalized for severe infection in low-and-middle-income countries sheds further light on these challenges. In low-and middle-income country studies, neonatal deaths were associated with prematurity, low birthweight and low postnatal age, similar to findings from high-income countries (19). In addition, absence of breastfeeding, malnutrition and respiratory or cardiovascular dysfunction were key risk factors. These findings may help to stratify patients most likely to benefit from preventive and targeted therapeutic interventions.

van Nassau et al. tested the accuracy of an age-adapted quick SOFA score (qSOFA) to predict the combined outcome of death and transfer to a PICU in children presenting to the ED for suspected bacterial infection requiring admission to the hospital. In their study, the proportion of children with critical illness requiring admission to a PICU and mortality were very low, and the prognostic accuracy of qSOFA, SIRS and qPELOD-2 was only modest. While the study cohort was small, the findings imply that current sepsis criteria do not perform sufficiently well to enable robust risk stratification of children with suspected bacterial infection. Novel approaches based on prospective collection of vital signs and laboratory values in large cohorts including electronic health records are urgently needed. In this context, it is important to consider that vital sign thresholds based on normal values according to age are incorporated in most recommendations and screening tools for risk stratification in the ED. However, normal values for vital signs of children so far were based on relatively small cohorts using traditional data capture methods. Sepanski et al. accessed a database of over 1 million medical records to provide a novel and reliable representation of heart rate and respiratory rate distribution in children presenting to the ED and who did not require hospitalization. This data will be useful to develop new risk scores and disease screening tools with increased sensitivity and specificity, to update current guidelines and improve alarm limits for bedside monitors.

In children, sepsis most commonly occurs in the community and the timing when parents seek medical care may influence disease severity and outcomes. In this context, Harley et al. reviewed the literature on the role of parental concerns in the recognition of sepsis in children and underscored the paucity of published data. Future studies are needed to develop evidence-based tools incorporating parental assessment of severity, parental decision-making in seeking medical care, and determine the diagnostic value of parental concerns.

## Sepsis Resuscitation

Early identification and appropriate resuscitation and management are critical to optimize outcome of children with sepsis. The Surviving Sepsis Campaign recently published updated guidelines for the management of septic shock in children (20). In past as well as in the present guidelines, fluid bolus therapy remains a first line cornerstone treatment for resuscitation of pediatric septic shock. Gelbart reviewed the current knowledge and challenges regarding fluid bolus therapy in pediatric sepsis. While clinical signs, echocardiography and other non-invasive and invasive monitoring tools are commonly used to assess shock and define circulatory status, the accuracy of these approaches to predict response to fluid boluses remains very limited. Given an increasing body of literature documenting potential harm related to excessive fluid therapy, restrictive fluid resuscitation protocols such as early vasoactive support needs to be investigated.

## Optimization of Antibiotic Use

Administering the right antibiotic, at the right dosage to the right patient and at the right time for the right duration remains a challenge toward optimal management of patients with suspected or proven infection. Antimicrobial stewardship (AMS) aims at improving the safe and appropriate use of antimicrobials, for better patient outcomes and reduction of antimicrobial resistance. Steinmann et al. reviewed the literature on the impact of leadership style on implementation and success of AMS and infection prevention programs, and share their own experience in a mixed NICU/PICU. A leadership style focused on empowering staff to take responsibilities led to higher engagement of staff and was associated with a reduction of antibiotic use and nosocomial infections.

van Donge et al. explored the complex relation between antibiotic regimen, exposure and response. Selecting the best antibiotic regimen is particularity challenging for neonates, due to rapid changes in drug metabolism and renal function during the first days and weeks of life which altogether alter drug distribution and elimination. Tauzin et al. presented a study on exposure to vancomycin in neonates receiving continuous drug infusion, and compared their results to those obtained using simulations with different models. This study highlights the challenges of prescribing a drug with a narrow therapeutic margin, the need for therapeutic drug monitoring and the importance of conducting pharmacokinetic studies.

Blood cultures remain a cornerstone of antibiotic stewardship to streamline targeted treatment and reduce unnecessary antibiotics. In most hospitals, children, and neonates with suspected sepsis are empirically treated for at least 48 h awaiting results of blood cultures, based on recommendations supported by limited evidence. Dierig et al. presented an analysis of blood-culture proven sepsis episodes in neonates and children included in the Swiss Pediatric Sepsis Study. In this prospective national cohort study, the median time to positivity, defined as the time between placement of the blood culture bottle into the automated system and a positive signal, was 12 h (IQR 8–17 h), and 88%, and 96%, of blood cultures were positive by 24 and 36 h, respectively. These findings indicate that the decision to continue empiric antibiotic treatment in the absence of positive blood culture should be reconsidered already after 24–36 h.

Culture-negative sepsis designates presumed symptomatic infection without a documented pathogen, and represents a substantial proportion of episodes in patients treated for presumed sepsis. Klingenberg et al. critically reviewed the entity of culture negative neonatal early-onset sepsis and propose strategies to improve AMS in early-life, without compromising efficient care.

Biomarkers are commonly used in the clinic to guide antibiotic treatment. In a prospective study conducted in two NICUs, Dillenseger et al. measured circulating levels of CRP, PCT, IL-6, and IL-8 at the time of clinical presentation, and evaluated their diagnostic performance to identify newborns with nosocomial sepsis. This study confirms previous studies showing that—across all age groups—biomarkers used alone or in combination have a limited value to help clinicians decide whether or not to initiate antibiotic treatment (21, 22).

Ventilator-associated pneumonia (VAP) is amongst the leading causes of nosocomial infection in intensive care units, and account for a large proportion of antibiotic use in NICUs and PICUs (23, 24). Diagnosis and confirmation of VAP is difficult in neonates, which may result both in overtreatment and delays in initiation of appropriate treatment with antibiotics. Goerens et al. presented the results of a quality improvement initiative for neonatal VAP. The intervention based on a prevention bundle and AMS interventions resulted in a decline in VAP incidence and antibiotic use.

## Epidemiology

The distribution of pathogens causing invasive infection evolves over time and is influenced by the practices used to prevent and treat infections. Epidemiological studies are important for benchmarking and quality improvement, to update policies and practices based on the most prevalent pathogens and their susceptibility to antibiotics, and to identify patients at the highest risk of developing infection and infection-related complications. Conjugated meningococcal vaccines have had a tremendous impact on reducing the incidence meningococcal sepsis and meningitis. Yet, *Neisseria meningitidis* remains a major agent causing sepsis and meningitis worldwide, and is associated with significant mortality, and long term disability in many survivors. Nadel and Ninis reviewed the preventive strategies, clinical features, and management of invasive meningococcal disease in the area of vaccination, highlighting the importance of detection and early management of the disease to improve patient outcome. Xu et al. reported on a cohort of term newborns with meningitis in Shanghai. Group B *Streptococcus* and *Escherichia coli* were the predominant pathogens. The high proportion of patients with abnormal neurological examination at discharge, abnormal magnetic resonance imaging and/or withdrawal of treatment underscores the considerable burden of disease. Furthermore, particular patient groups are much more susceptible to sepsis, as illustrated by patients with sickle cell disease. Increased blood viscosity and vascular occlusion result in functional asplenia and immune deficiency, thereby increasing susceptibility to bacterial infections. Ochocinski et al. reviewed the life-threatening infectious complications of sickle cell disease, and identified priorities for prevention and treatment of infections in high- and low-income countries.

Toxic Shock Syndrome (TSS) is a severe acute illness caused by toxin-producing strains of *Staphylococcus aureus* or *Streptococcus pyogenes*. The study by Javouhey et al. shows that *Staphylococcus* and *Streptococcus* TSS in children differ by their source of infection, clinical presentation, disease severity and outcome. *Staphylococcus* TSS predominantly originated from the female genital tract, while *Streptococcus* TSS was associated with pulmonary infection and bacteremia, a more frequent occurrence of respiratory failure and a longer duration of mechanical ventilation and stay in PICU.

Bacterial and fungal infections are most commonly attributed as the cause of sepsis. However, viruses can trigger dysregulated host responses, leading to life-threatening organ dysfunction as illustrated by the current COVID-19 pandemic. Gupta et al. summarized the epidemiology, pathophysiology and diagnostic and therapeutic aspects of the management of viral sepsis. The importance of early recognition and pathogen identification in viral sepsis has important implications for AMS, infection control measures, risk stratification, and in some cases antiviral therapies.

## Conclusions

The striking impact of sepsis on child health indicates that developmental aspects such as mode of transmission, pathogen susceptibility, and host response, underpin the epidemiology of childhood sepsis. A better understanding of the heterogeneity of the disease, age-specific epidemiology and pathophysiology remains a key requirement to prevent sepsis and reduce disease severity, improve short and long term outcomes, and lessen the burden for the society. To date, populational data still compare predominantly “count” data on sepsis cases and sepsis mortality, failing to take into account that sepsis in children affects patients with a life expectancy of up to 85 years, leading to a disproportional impact on quality adjusted life years and years of life lost. There is urgency for future studies to reflect on the whole-of-life and whole-of-society impact of pediatric sepsis integrating mortality, morbidity, long-term outcomes (25), and direct and indirect costs.

The collection of articles on sepsis in this Frontiers Topic highlights our current understanding, knowledge gaps and limitations of approaches to prevent, diagnose, and treat sepsis, and priorities for future research.

Key areas emerging as future research priorities include first, prevention though enhanced hygiene, modulation of the microbiome and nutritional strategies, and vaccines. Second, there is a major need to better define the biological and clinical phenotypes of neonates and children with sepsis enabling reliable discrimination between children with uncomplicated infection and those where infection leads to organ dysfunction due to dysregulated host response. Third, deciphering the heterogeneity of sepsis in neonates and children will enable novel approaches for targeted individualized interventions more likely to change disease trajectories in individual patients. The increasing availability of biological (OMICs) and high resolution clinical data from electronic health records and the rapid progress in applying computational science to health data is likely to change our approach to sepsis in children in the coming decades. Integrative approaches may enhance clinical evaluation at the bedside, and enable the development of artificial intelligence-based sepsis recognition and risk stratification tools with the ultimate view to deliver precision medicine. Forth, currently available observational data indicate that the most substantial outcome improvements in sepsis can be achieved by reliable implementation of systems to systematically screen and treat children with sepsis aiming to enhance reliability of sepsis care across health care systems (3, 26). Finally, it is imperative that sepsis campaigns work hand in hand with AMS initiatives to reduce unnecessary exposure to antibiotics in children which do not suffer from bacterial infections (5).

## Author Contributions

All authors listed have made a substantial, direct and intellectual contribution to the work, and approved it for publication.

## Conflict of Interest

The authors declare that the research was conducted in the absence of any commercial or financial relationships that could be construed as a potential conflict of interest.
